# Targeting ORM1-CCR5 axis inhibits the aggressive phenotype of synovial fibroblasts and alleviates RA

**DOI:** 10.1515/med-2025-1365

**Published:** 2026-01-13

**Authors:** Min Liu, Yutong Chen, Xuyang Ding, Linling Luo, Yining Xu, Xide Liu

**Affiliations:** Department of Traditional Chinese Medicine, Zhejiang Hospital of Integrated Traditional Chinese and Western Medicine, Hangzhou, Zhejiang, China; The Second School of Clinical Medicine, Zhejiang Chinese Medical University, Hangzhou, Zhejiang, China

**Keywords:** rheumatoid arthritis, orosomucoid 1, C-C chemokine receptor 5, synovial hyperplasia, fibroblast-like synoviocytes

## Abstract

**Objectives:**

The present study focused on exploring the role of orosomucoid 1 (ORM1) in rheumatoid arthritis (RA).

**Methods:**

Differentially expressed genes in GSE15573 dataset were analyzed by bioinformatics. Fibroblast-like synoviocytes (FLS) from RA patients were stimulated with collagen-II, followed by quantification of ORM1 and C-C chemokine receptor 5 (CCR5) expressions. The interaction between ORM1 and CCR5 in FLS was examined by Co-immunoprecipitation. After ORM1 intervention or maraviroc treatment, the effect of ORM1 or CCR5 as well as their interplay in the stimulated cells was investigated using molecular experiments, methylthiazolyldiphenyl-tetrazolium bromide assay and transwell assay. Rats underwent collagen-induced arthritis (CIA) modeling. Arthritis index scoring, western blot assay and histopathological evaluation were performed in CIA rats with ORM1 or CCR5 knockdown.

**Results:**

ORM1 and CCR5 were highly expressed in collagen type II (CII)-stimulated FLS and synovial tissues of CIA rats. The expression of CCR5 was positively regulated by ORM1, and the interaction of ORM1 and CCR5 was confirmed. CII stimulation enhanced viability, migration and invasion of FLS, but these effects were antagonized in the absence of ORM1 or CCR5. Knockdown of ORM1 or CCR5 in CIA rats reduced arthritis index, while alleviating cartilage erosion, inflammatory infiltration and synovial hyperplasia of ankle joints.

**Conclusions:**

ORM1 deficiency suppresses the aggressive phenotype of FLS to reduce RA progression by downregulating CCR5.

## Introduction

Rheumatoid arthritis (RA) is a systemic autoimmune disease that predominantly involves the joints, and is characterized by synovial hyperplasia, progressive cartilage and bone damage as well as extra-articular manifestations [1], 2]. The latest epidemiological data showed that AR affects 0.27 % of the global population and its development is closely associated with genetics and environmental risk factors [3], 4]. Accumulating evidence has demonstrated that tissue destruction in RA manifests as synovitis, which is triggered and maintained by the interaction of multiple cells, resulting in the synovial membrane to expand and invade the periarticular bone [5], 6]. Although significant progress has been made in lowering disease activity and mortality of RA, a majority of patients still suffer from functional disability [7]. Therefore, further elucidation of the pathological mechanism underlying RA is essential to improve disease management.

Orosomucoid (ORM), an acute response protein, is produced by a variety of human tissues under physiological and pathological conditions, and participates in many biological activities, such as immunomodulation, protein transport and maintenance of capillary function [8]. ORM1 is frequently found to be upregulated in the serum during inflammation [9]. ORM1 also plays a part in the development of infection, kidney injury and cancer [10], [11], [12]. Reportedly, ORM1 is abnormally expressed in osteoarthritis and might be involved in the structural remodeling of subchondral bone [13]. Although ORM1 expression has been associated with RA disease activity, its specific role in RA progression remains largely unexplored.

C-C chemokine receptor 5 (CCR5) is a transmembrane G-protein coupled receptor and is expressed on various cells, such as immune cells, osteoclasts, fibroblasts and cancer cells [14]. It has been widely reported that CCR5 contributes to inflammatory diseases and tumorigenesis by binding to multiple ligands [15], 16]. The expression of CCR5 is associated with the severity of RA, and downregulation of CCR5 mitigates joint inflammation in collagen-induced arthritis (CIA) mice through the regulation of immune responses [17]. CCR5 has been recently identified as a membrane receptor of ORM1, deletion of which can inhibit the anti-fatigue role of ORM1 in muscle cells [18].

In the present study, we focused on investigating the effect of ORM1 on the development of RA *in vitro* and *in vivo*, as well as further revealing whether CCR5 is implicated in the regulatory mechanism of ORM1, with the aim to provide new therapeutic strategies for RA.

## Methods

### Biopsy sample collection and ethics statement

Synovial tissues from RA patients were sampled during the surgery of knee replacement at Hangzhou Red Cross Hospital. This study was performed in accordance with the tenets of the Helsinki Declaration and was approved by the Ethics Committee of Hangzhou Red Cross Hospital ([2023] Research Review No. 117) after obtainment of each participant’s written informed consent.

### Animals

Six-week-old Sprague Dawley (SD) rats (male, 170–210 g) were housed in a specific pathogen-free environment with food and drinking water *ad libitum*. All experiments involving animals in this study have been approved by the Ethics Committee of Zhejiang Baiyue Biology Technology Co., Ltd. (Approval No. ZJBYLA-IACUC-20230608), and all experimental procedures were conducted based on the guidelines of the China Council on Animal Care and Use.

### Bioinformatics analysis

A GSE15573 dataset from the Gene Expression Omnibus (GEO) database (https://www.ncbi.nlm.nih.gov/geo/) was retrieved to analyze differentially expressed genes in peripheral blood mononuclear cells (PBMCs) from 18 RA patients and 15 controls using the multiple linear regression limma package. The cut-off values for the screening of differentially expressed genes were set as |log2 fold change| >1 and adjusted p-value <0.05. DAVID database (https://david.ncifcrf.gov/) was employed for the functional and pathway enrichment analysis of differentially expressed genes, including biological processes (BP), cellular components (CC), molecular functions (MF) and Kyoto Encyclopedia of Genes and Genomes (KEGG) pathway. Results were read using “R program” (version: R 4.3).

### Isolation and culture of primary fibroblast-like synoviocytes (FLS)

FLS isolation was performed as described previously [19]. In brief, synovial biopsy tissues were dissected to remove adipose tissues and rinsed with α minimum essential medium (α-MEM; 12561056, Thermo Fisher, Waltham, MA, USA). Next, cartilage tissues were minced and digested in α-MEM supplemented with 1 mg/mL collagenase I (17100017, Thermo Fisher, USA) at 37 °C for 6 h. After filtration and centrifugation, cells were suspended in α-MEM containing 20 % fetal bovine serum (FBS; S4115, Biochrom AG, Berlin, Germany) and 1 % penicillin-streptomycin solution (C0222, Beyotime, Shanghai, China). FLS were cultured in a 5 % CO_2_ incubator at 37 °C after identification of flow cytometry.

### Cell treatment

FLS at passage three were stimulated with collagen type II (CII; C8000, Solarbio, Beijing, China) at 37 °C for 6 h. To explore whether CCR5 participates in the RA-promoting effect of ORM1, maraviroc (MVC; a CCR5 antagonist) solution (S2003, Selleck Chemicals, Houston, TX, USA) was diluted with the culture medium at doses of 5, 10, 20 and 40 μM [20]. FLS in 96-well plates (3×10^3^ cells/well) were cultured overnight and treated with or without different doses of MVC at 37 °C for 24 and 48 h, respectively. Then, 10 μL cell counting kit-8 reagent (CK04, Dojindo, Tokyo, Japan) was added in each well for 3-h incubation. Absorbance detection (450 nm) was performed using a microplate reader (Spark, Tecan, Männedorf, Switzerland) to analyze the cytotoxicity of MVC.

### Cell transfection

Small interfering RNA (siRNA) targeting ORM1 (SiORM1; sense: 5′-UCA​AAA​GCA​AGC​AUG​UAG​GUC-3′, antisense: 5′-CCU​ACA​UGC​UUG​CUU​UUG​ACG-3′), as well as siRNA negative control (SiNC) was customized from GenePharma (Shanghai, China). FLS were seeded in 6-well plates at a density of 1.5×10^5^ cells/well and then transfected with indicated siRNA at 37 °C using Lipofectamine RNAi Max (13778030, Thermo Fisher, USA) following the manufacturers’ protocol. After 48 h of transfection, the cells underwent the verification of transduction efficiency using quantitative real-time reverse transcription polymerase chain reaction (qRT-PCR).

### RNA isolation and qRT-PCR

After cell treatment or/and transfection, FLS were homogenized in TRI reagent (T9424, Sigma-Aldrich, St Louis, MO, USA), followed by 15-min centrifugation (12,000×*g*) at 4 °C to isolate total RNA. Next, RNA was reverse-transcribed into complementary DNA using Color Reverse Transcription Kit (A0010CGQ, EZBioscience, Roseville, MN, USA), and PCR was carried out with primers and 2×Color SYBR Green qPCR Master Mix (A0012-R2, EZBioscience, USA) in a qRT-PCR system (4471087, Applied Biosystems, Carlsbad, CA, USA). Relative mRNA expressions of ORM1 and CCR5 were calculated using the 2^−ΔΔCt^ method [21], and data were normalized to the endogenous control of GAPDH. Primer sequences used in the reaction are listed as follows (5′-3′): ORM1 (forward: ACA​CCA​CCT​ACC​TGA​ATG​TCC, reverse: GTG​AGC​GAA​ATG​CTC​TTG​GC), CCR5 (forward: TTC​TGG​GCT​CCC​TAC​AAC​ATT, reverse: TTG​GTC​CAA​CCT​GTT​AGA​GCT​A) and GAPDH (forward: TGT​AGG​CTC​ATT​TGC​AGG​GG, reverse: TCC​CAT​TCC​CCA​GCT​CTC​AT).

### Methylthiazolyldiphenyl-tetrazolium bromide (MTT) assay

After cell treatment or/and transfection, FLS were plated at a density of 3×10^3^ cells/well and cultured overnight, followed by 4-h incubation with 10 μL MTT (298-93-1, Yuanye Bio-Technology Co., Ltd, Shanghai, China). Then, dimethyl sulfoxide (ST038, Beyotime, China) was utilized to dissolve the formazan product in cells. Results were detected at 570 nm by a microplate reader. Cell viability was calculated as follows: Cell viability (%) = (A_experimental_ – A_blank_)/(A_control_ – A_blank_) ×100 %.

### Transwell assay

The invasion or migration of FLS receiving cell treatment or/and transfection was evaluated using transwell assay with transwell supporters (3462, 24-well format, Corning Inc., Corning, NY, USA) pre-coated with/without matrigel (356234, Corning Inc., USA). Briefly, 1×10^4^ cells in 100 µL serum-free medium were plated in the upper chamber of transwell supporters, and the lower chamber was filled with 600 µL FBS-containing medium, followed by 48-h incubation at 37 °C. Afterwards, the supporters were fixed with 4 % paraformaldehyde (PFD; P39200, Acmec Biochemical, Shanghai, China) and stained with 0.1 % crystal violet (G1064, Solarbio, China) at room temperature, in sequence. To minimize observational bias, all subsequent counting procedures were performed under blinded conditions: each membrane was assigned a random code by an independent investigator not involved in the counting process. The stained membranes were imaged using a light microscope (Primovert, ZEISS, Lena, Germany) at ×250 magnification. Five fields of view were randomly selected and captured on each membrane, where the number of transmembrane cells were counted using Image J software v2.0.0 (NIH, Bethesda, MD, USA). The average count was calculated and used for further statistical analysis. Relative migration rate (%) = (Number of migrating cells in the treatment group/Number of migrating cells in the control group)×100; Relative invasion rate (%)=(Number of invasive cells in the treatment group/Number of invasive cells in the control group)×100.

### Animal experiment and assessment of arthritis

Lentivirus expressing short hairpin RNAs (shRNAs) targeting ORM1 (lenti-ORM1-KD) or CCR5 (lenti-CCR5-KD) were constructed using Lv-GFP/puro vectors and provided by Public Protein/Plasmid Library (Nanjing, China). CIA was constructed as instructed [22]. In brief, 2 mg/mL chicken CII (C9301, Sigma-Aldrich, USA) was prepared with glacial acetic acid (F-036289, Acmec Biochemical, China) and then, was mixed with Freund’s complete adjuvant (FCA; P2036, Beyotime, China) to obtain CII/FCA emulsion. SD rats were randomly divided into four groups (n=5 rats/group): Con group, CIA+lenti-NC group, CIA+lenti-ORM1-KD group and CIA+lenti-CCR5-KD group. CIA rats were modeled by injecting 0.1 mL emulsion separately into the tail root and paws. Then, a second injection with 0.1 mL emulsion was conducted on the tail root of each rat after 7 days. After modeling, rats from the latter three groups were injected with Lv-shNC-GFP/puro vectors, lenti-ORM1-KD and lenti-CCR5-KD (3×10^5^ transduction unit) into the joint cavity, respectively, on day 18. The lentiviral injection was performed once a week for 4 weeks. At the end of the experiment, photos of rat paw and arthritis index were recorded. Arthritis index was scored on a scale of 0–4, with 0=no redness, 1=mild swelling of the ankle, 2=moderate swelling of the ankle, 3=severe swelling of the ankle, and 4=swelling of the entire foot walking difficulty [23]. All rats were euthanatized by cervical dislocation under deep anesthesia (150 mg/kg pentobarbital sodium, P3761, Sigma-Aldrich, USA). Ankle joints and synovial tissues were harvested from the left hindlimb of rats for following assays.

### Western blot

FLS or rat synovial tissues were homogenized in phenylmethanesulfonyl fluoride-contained RIPA buffer (R0020, Solarbio, China) on ice to extract total protein. The lysed samples were subjected to 5-min centrifugation (14,000×*g*) to collect supernatant, and then protein quantification was performed with BCA Protein Assay Kit (T9300A, Takara, Beijing, China). 20 mg of protein samples were loaded in 10 % SDS-PAGE gel under denaturing conditions, electrophoretically transferred to polyvinylidene fluoride membranes (FFP20, Beyotime, China), and treated with blocking buffer (P0252, Beyotime, China) at room temperature for 15 min. Primary antibodies against ORM1 (ab200732, 24 kDa, 1:1,000, Abcam, Cambridge, UK), CCR5 (A20261, 41 kDa, 1:1,000, ABclonal, Wuhan, China) and endogenous control GAPDH (ab8245, 37 kDa, 1:10,000, Abcam, UK) were utilized to incubate the separated blots on the membranes at 4 °C overnight, after which the incubation with corresponding horseradish peroxidase-conjugated secondary antibodies (AS014/AS003, ABclonal, China) was carried out at room temperature for 2 h. At length, the blots were visualized with chemiluminescence solution (P1000, Pplygen, Beijing, China) in a Quantity One System image analyzer (Bio-Rad, Hercules, CA, USA). The protein expression was normalized relative to GAPDH expression. Relative protein expression = grey value of each protein/grey value of GAPDH.

### Co‐immunoprecipitation

Total protein was extracted from FLS and subjected to co-immunoprecipitation using IP/CO‐IP Kit (Thermo Fisher Scientific) according to the manufacturer’s instructions. All steps were performed at 4 °C. To be specific, each protein sample from the selected groups was aliquoted into its own centrifuge tube, followed by the addition of 1 µg rabbit IgG or 1 µg corresponding immunoprecipitating antibodies, and then incubated overnight. Following a 1-h incubation with 20 µL Protein A/G beads, the samples were centrifuged and the supernatant was discarded. The Protein A/G beads were washed twice with 1 mL lysis buffer, with the supernatant being removed after each centrifugation. Later, the Protein A/G beads were resuspended in 5× loading buffer and boiled for 5 min. Finally, the supernatant was collected and transferred to a new tube for Co-immunoprecipitation with the antibody against ORM1 (ab200732, 1:1,000, Abcam, Cambridge, UK) and CCR5 (A20261, 1:1,000, ABclonal, Wuhan, China). The CCR5 and ORM1 protein expression was used by Western blot analysis.

### Histopathological evaluation

Rat ankle joints were fixed with 4 % PFD for one week before decalcification with ethylene diamine tetraacetic acid solution (R20403, Yuanye Bio-Technology Co., Ltd, China). Following dehydration and paraffin embedding, the joint samples were sectioned into 5 μm-thick slices. To evaluate cartilage erosion, the slices were subjected to Safranin O-fast green staining according to the manufacturers’ protocol of Modified Saffron-O and Fast Green Stain Kit (G1371, Solarbio, China). To examine pathological changes of the ankle joints, the slices were stained with hematoxylin (C0107, Beyotime, China) and eosin (C0109, Beyotime, China) at room temperature, in sequence. Images of cartilage erosion and pathological changes at ×40 magnification were observed by light microscopy, followed by scoring analysis [24].

### Statistical analysis

Data from at least three independent experiments are expressed as mean±standard error of the mean, and analyzed using GraphPad Prism v8.0 (GraphPad Software Inc., San Diego, CA, USA). Comparisons among multiple groups and between two groups were analyzed using one-way analysis of variance and independent samples t-test, respectively. Difference with a p-value of <0.05 suggested a statistical significance.

### Ethical approval

This study was performed in accordance with the tenets of the Helsinki Declaration and was approved by the Ethics Committee of Hangzhou Red Cross Hospital ([2023] Research Review No. 117) after obtainment of each participant’s written informed consent.

All experiments involving animals in this study have been approved by the Ethics Committee of Zhejiang Baiyue Biology Technology Co., Ltd. (Approval No. ZJBYLA-IACUC-20230608), and all experimental procedures were conducted based on the guidelines of the China Council on Animal Care and Use.

## Results

### Identification of ORM1 in RA

**Figure 1:**
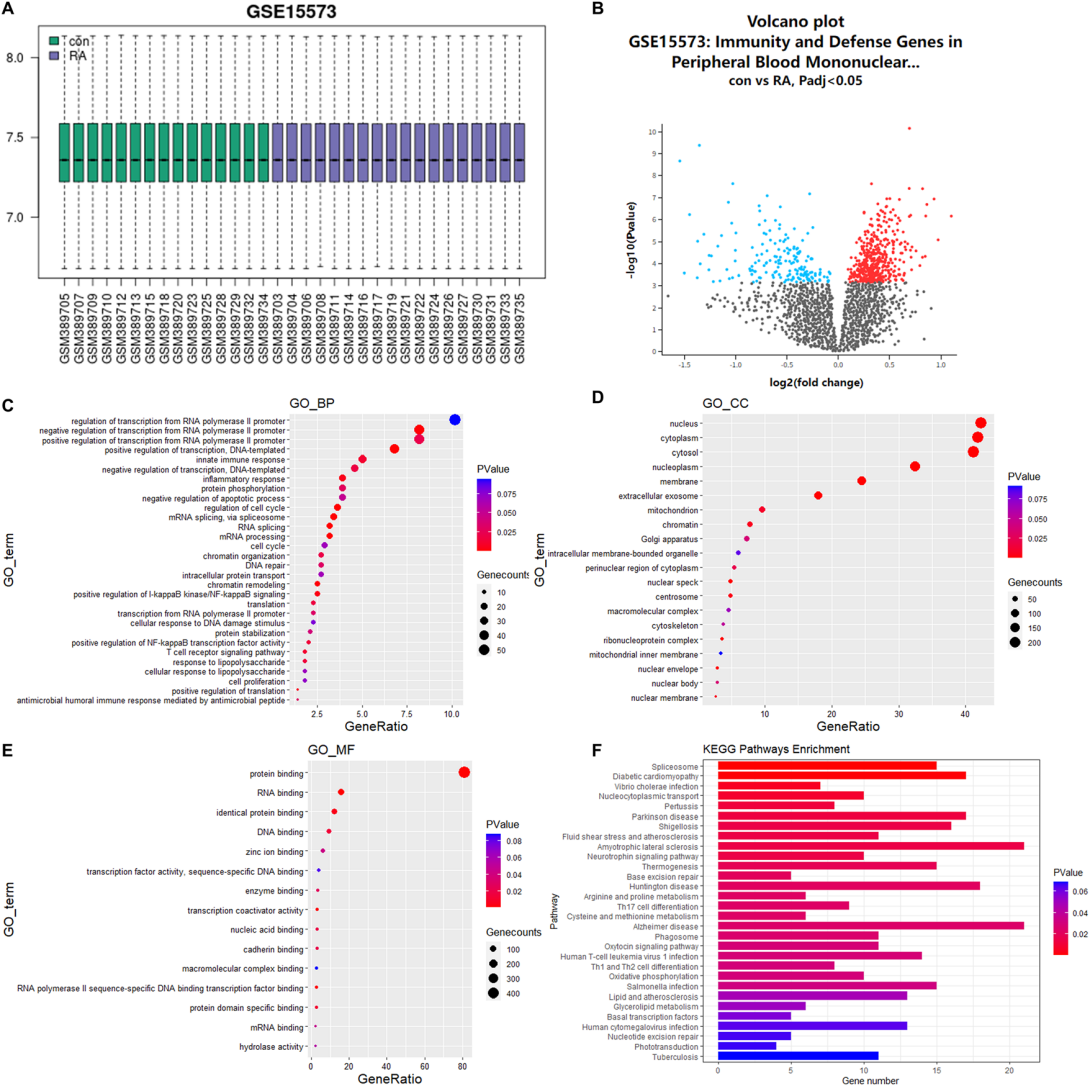
Analysis of differentially expressed genes associated with RA. (A) Normalization of total differentially expressed genes in a GSE15573 dataset. (B) Volcano plot of differentially expressed genes in peripheral blood mononuclear cells of RA. (C–F) BP, CC, MF and KEGG pathway enrichment analysis of differentially expressed genes by DAVID database. Abbreviation: RA, rheumatoid arthritis; BP, biological processes; CC, cellular components; MF, molecular functions; GO, gene ontology; KEGG, kyoto encyclopedia of genes and genomes.

The data of gene expression in GSE15573 dataset were normalized and presented in a boxplot (Figure 1A). According to the established criteria, we identified differentially expressed genes in PBMCs of RA patients, and ORM1 was found to be upregulated, as presented in a volcano plot (Figure 1B). According to Figure 1C, the results of Gene Ontology (GO) analysis showed that in the BP ontology, the identified differentially expressed genes were involved in negative regulation of transcription from RNA polymerase II promoter and inflammatory response. In the CC ontology, the identified differentially expressed genes were enriched in nucleus, cytoplasm, cytosol, nucleoplasm and membrane (Figure 1D). In the MF ontology, the identified differentially expressed genes were mainly enriched in protein binding (Figure 1E). Besides, top 30 KEGG pathways were shown in Figure 1F, and the identified differentially expressed genes were confirmed to be mainly associated with amyotrophic lateral sclerosis, Alzheimer disease, Huntington disease and diabetic cardiomyopathy.

### CCR5 was highly expressed in CII-stimulated FLS and its expression was regulated by ORM1

**Figure 2:**
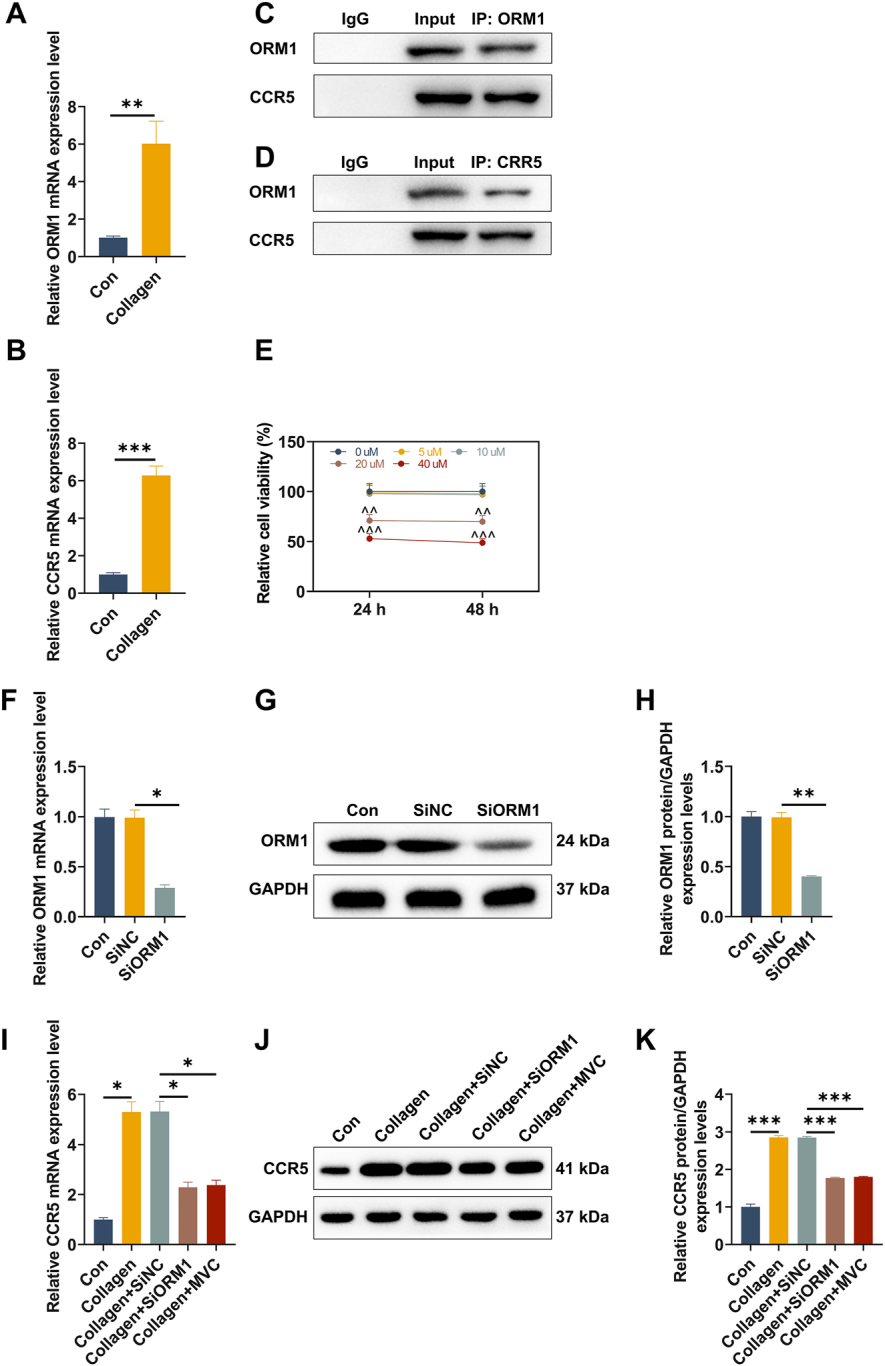
Expressions of ORM1 and CCR5 in CII-stimulated FLS as well as their interaction. (A–B) RA-derived FLS were stimulated with or without CII for 6 h, and qRT-PCR was performed to detect the mRNA expressions of ORM1 and CCR5. (C–D) The interaction of ORM1 and CCR5 in FLS were examined by Co-immunoprecipitation experiment. (E) RA-derived FLS were treated with 0, 5, 10, 20 and 40 μM MVC (a CCR5 antagonist) for 24 and 48 h, and cell counting kit-8 was used to detect cell viability. (F) RA-derived FLS were transfected with/without SiNC or SiORM1, and qRT-PCR was performed to verify transduction efficiency. (G–H) RA-derived FLS were transfected with/without SiNC or SiORM1, and western blot was conducted to verify transduction efficiency. (I) RA-derived FLS were subjected to SiNC/SiORM1 transfection, CII stimulation or MVC treatment (con, collagen, Collagen+SiNC, Collagen+SiORM1 and Collagen+MVC groups), and the mRNA expression of CCR5 in each group was determined by qRT-PCR. (J–K) the protein expression of CCR5 in each group was measured by western blot. Data are shown as mean±standard deviation. GAPDH was used as the endogenous control. ^*^p<0.05, ^**^p<0.01, ^***^p<0.001. Abbreviation: FLS, fibroblast-like synoviocytes; CII, collagen type II; ORM1, orosomucoid 1; CCR5, C-C chemokine receptor 5; MVC, maraviroc; SiORM1, small interfering RNA (siRNA) targeting ORM1; SiNC, siRNA negative control; qRT-PCR, quantitative real-time reverse transcription polymerase chain reaction.

Compared with normally cultured FLS, the expressions of ORM1 and CCR5 were elevated in CII-stimulated FLS (Figure 2A and B, p<0.01). In addition, the interaction of ORM1 and CCR5 was confirmed in FLS (Figure 2C and D). To inhibit the expression of CCR5 in FLS, we treated the cells with 0, 5, 10, 20 and 40 μM MVC for 24 and 48 h, respectively. As shown in Figure 2E, MVC at 20 and 40 μM presented the significant cytotoxicity (p<0.01). Thus, 24-h treatment with 10 μM MVC was used for subsequent *in-vitro* experiments. After transfection with siRNA, qRT-PCR and western blot data determined that the expression of ORM1 was decreased in SiORM1-transfected FLS relative to SiNC-transfected FLS (Figure 2F–H, p<0.05). Compared with CII-stimulated FLS, SiORM1 reduced the expression of CCR5 in the cells (Figure 2I–K, p<0.05). Of note, similar results were discerned in CII-stimulated FLS after MVC treatment (Figure 2I–K, p<0.05). These findings implied a regulatory effect of ORM1 on CCR5 in RA.

### Inhibition of ORM1 or CCR5 reduced viability, migration and invasion of CII-stimulated FLS

**Figure 3:**
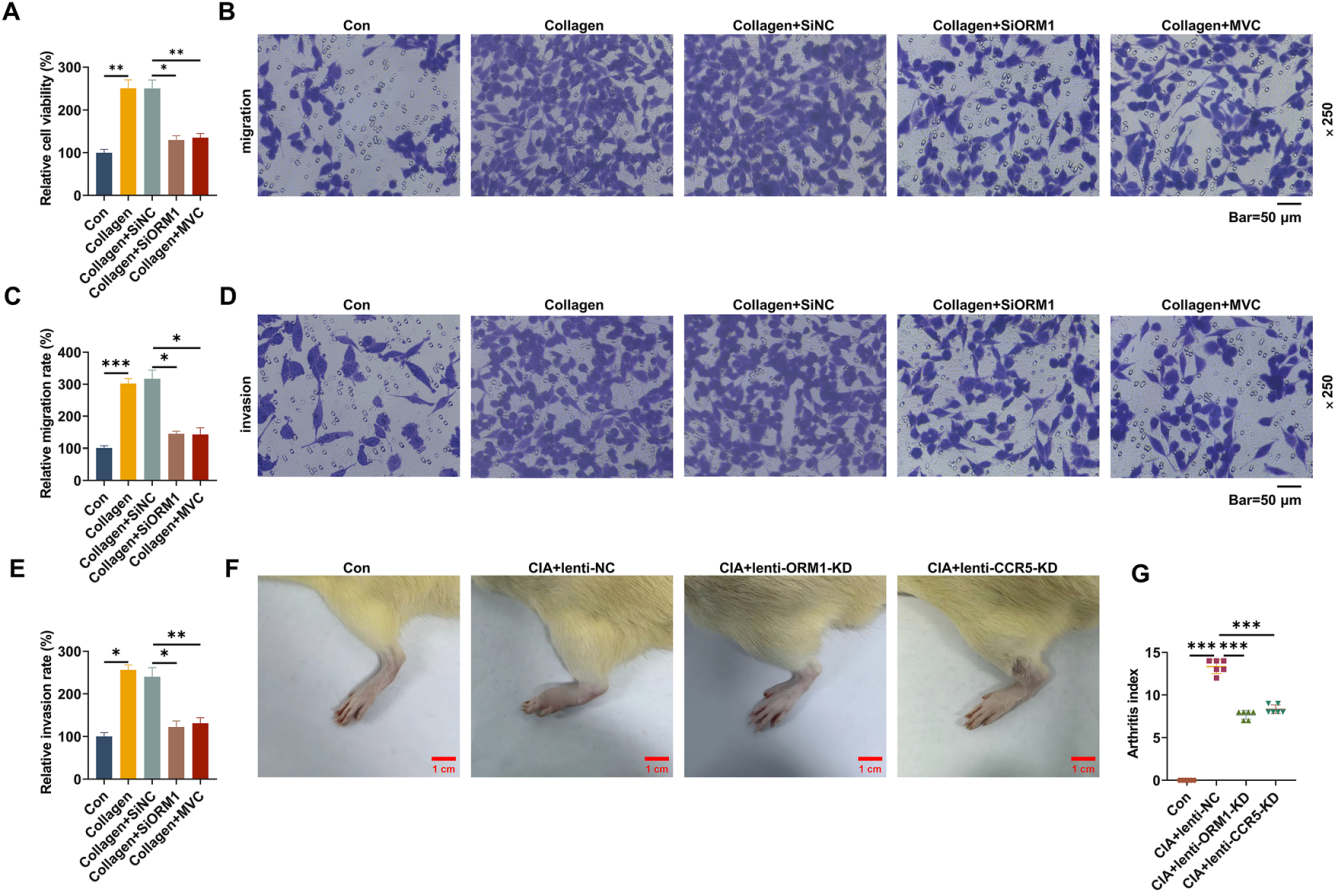
Effects of ORM1 and CCR5 on CII-stimulated FLS migration/invasion and CIA arthritis. (A) RA-derived FLS were subjected to SiNC/SiORM1 transfection, CII stimulation or MVC treatment (con, collagen, Collagen+SiNC, Collagen+SiORM1 and Collagen+MVC groups), and cell viability in each group was examined by methylthiazolyldiphenyl-tetrazolium bromide assay. (B–E) Representative image of migrating and invading cells in each group (magnification: ×250, scale bar=50 µm). (F) Sprague dawley rats underwent CIA modeling or not, followed by lentiviral injection on day 18 for 4 weeks with once per week (con, CIA+lenti-NC, CIA+lenti-ORM1-KD and CIA+lenti-CCR5-KD groups). Representative image of rat paw swelling was photographed in each group (scale bar=1 cm). (G) The arthritis index of rats was scored in each group. Data are shown as mean±standard deviation. ^*^p<0.05, ^**^p<0.01, ^***^p<0.001. Abbreviation: CIA, collagen-induced arthritis; lenti-ORM1-KD, lentivirus expressing ORM1 short hairpin RNA; lenti-CCR5-KD, lentivirus expressing CCR5 short hairpin RNA; lenti-NC, lentivirus expressing shNC.

As demonstrated in Figure 3A–E, CII stimulation enhanced viability, migration and invasion of FLS (p<0.05). Notably, ORM1 downregulation led to reduced cell viability, migration, and invasion (Figure 3A–E, p<0.05), which attenuated the effects of CII. After MVC treatment, the promoting effects of CII on viability, migration and invasion of FLS were also reversed (Figure 3A–E, p<0.05).

### Knockdown of ORM1 or CCR5 in CIA rats alleviated inflammation, cartilage erosion and synovial hyperplasia of ankle joints

**Figure 4:**
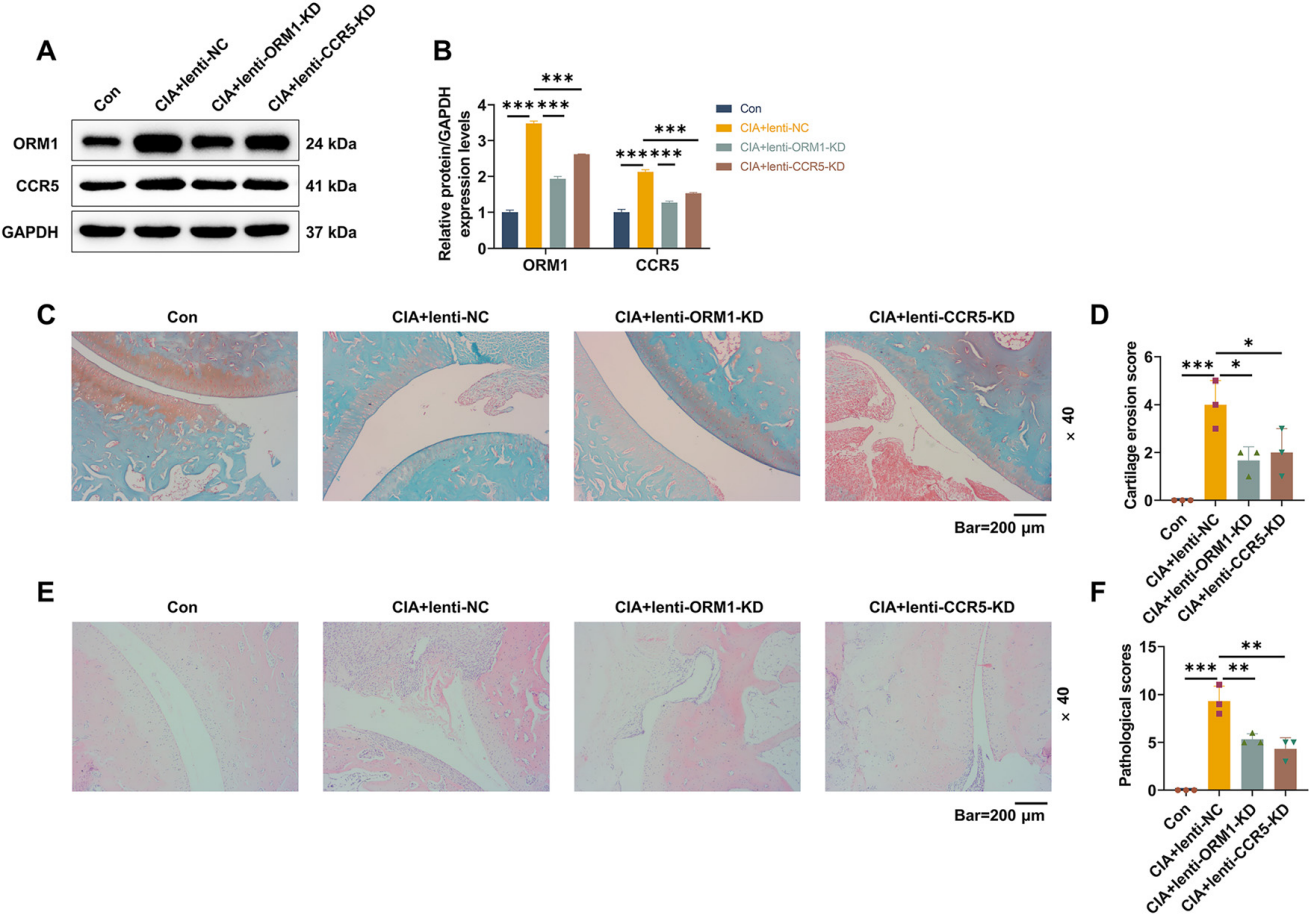
Effects of ORM1/CCR5 knockdown on cartilage erosion in CIA rats. (A–B) Sprague dawley rats received CIA modeling or not, followed by lentiviral injection on day 18 for 4 weeks with once per week (con, CIA+lenti-NC, CIA+lenti-ORM1-KD and CIA+lenti-CCR5-KD groups). Protein expressions of ORM1 and CCR5 in synovial tissues from each group were examined by western blot. (C) Representative image of safranin O-fast green staining of rat ankle tissues in each group (magnification: ×40, scale bar=200 µm). (D) The cartilage erosion of rats was scored in each group. (E) Representative image of hematoxylin and eosin staining of rat ankle tissues in each group (magnification: ×40, scale bar=200 µm). (F) The pathological changes of rats were scored in each group. Data are shown as mean±standard deviation. GAPDH was used as the endogenous control. ^*^p<0.05, ^**^p<0.01, ^***^p<0.001.

To further verify the mechanism of ORM1, we constructed CIA rats and treated them with or without lentiviral injection. As illustrated in Figure 3F and G, CIA rats developed significant paw swelling with increased arthritis index (p<0.001). Following injection with either lenti-ORM1-KD or lenti-CCR5-KD for 4 weeks, the swollen paw and the arthritis index of CIA rats were apparently alleviated (Figure 3F and G, p<0.001). Compared with normal rat synovial tissues, ORM1 and CCR5 expressions were increased in CIA rats-derived synovial tissues, as determined by western blot (Figure 4A and B, p<0.001). The lower expressions of ORM1 and CCR5 were detected in both CIA+lenti-ORM1-KD and CIA+lenti-CCR5-KD groups in contrast with CIA+lenti-NC group (Figure 4A and B, p<0.001). The results from Safranin O-fast green staining revealed that CIA modeling led to the cartilage erosion of rat ankle joints (Figure 4C and D, p<0.001), which was mitigated after knockdown of either ORM1 or CCR5 (Figure 4C and D, p<0.05). In addition, the results of hematoxylin and eosin staining demonstrated that CIA modeling promoted pathological changes of rat ankle joints, manifested by pronounced inflammatory infiltration and synovial hyperplasia (Figure 4E and F, p<0.001). The injection with lenti-ORM1-KD or lenti-CCR5-KD reduced inflammatory infiltration and synovial hyperplasia of ankle joints in CIA rats (Figure 4E and F, p<0.01).

## Discussion

Recently, ORM1 has been reported to be a novel biomarker of RA due to its positive correlation with immune infiltration and disease activity, suggesting its potential in the diagnosis and treatment of RA [25]. Herein, the deficiency of ORM1 reduced migration and invasion of FLS upon CII stimulation, which mechanistically was mediated by decreasing the expression of CCR5. Furthermore, it was verified that knockdown of ORM1 or CCR5 hindered the disease progression of CIA rats by ameliorating inflammation, cartilage erosion and synovial hyperplasia.

During the evolution of RA, the transformation of synovium into proliferative, invasive tissue, a major cause of cartilage and bone damage, is related to the phenotypic alteration of FLS and immune cell recruitment [26]. FLS form the intimal lining layer of the synovium and play a critical role in lubrication and nourishment of the cartilage surfaces at the joint junction by producing the synovial fluid and maintaining extracellular matrix homeostasis [27]. It is established that FLS present an aggressive phenotype and secret pathogenic mediators in RA; therefore, ameliorating the dysfunction of FLS is considered as a an attractive therapeutic strategy for restoring synovial homeostasis [28]. Previously, Zhao et al. have found that discoidin domain receptor two is overexpressed in CII-stimulated FLS, and interacts with annexin A2 to promote cartilage degradation by inducing proliferation, invasion and matrix metalloproteinases (MMPs) secretion of FLS [19]. The study of Najm et al. has demonstrated that blocking the JAK/STAT pathway reduces FLS-secreted pro-inflammatory cytokines including IL-6 and thereby alleviates inflammation and bone destruction in CIA mice [29]. According to bioinformatics analysis in this study, the upregulation of ORM1 was found in PBMCs of RA patients. Besides, the results of genetic function annotation revealed that the differentially expressed genes including ORM1 might be involved in immune response and inflammatory response, and be related to neurological disorders. As an immunity-related gene, ORM1 has been shown to affect macrophage polarization to mediate immune tolerance, thereby promoting the malignant phenotype of cancer cells [30], 31]. This study found that the expression of ORM1 was increased in RA-derived FLS after CII stimulation, as well as in synovial tissues of CIA rats. Downregulation of ORM1 was found to suppress the facilitating effects of CII on cell migration and invasion, as well as to reduce arthritis index, cartilage erosion, inflammatory infiltration and synovial hyperplasia of ankle joints in CIA rats.

High levels of chemokines and their receptors have been detected in PBMCs and synovial fluid of patients with RA [32]. The binding of chemokines and receptors can boost the accumulation of immune cells in synovial tissues and contribute to chronic inflammation [33]. Herein, we detected upregulation of CCR5 in CII-stimulated FLS and synovial tissues of CIA rats. Also, an existing study has identified upregulated CCR5 in synovial tissues of patients with RA, which acts as a hub gene in the development of RA through chemokine signaling pathway [34]. Nonetheless, the effect of CCR5 antagonists on RA is controversial [35]. The study of Ansari et al. has shown that administration with a CCR5 antagonist MVC reduces the progression of RA in CIA mice by suppressing inflammatory mediators and Th17 cell-related signaling [17]. In this study, we treated FLS with MVC for 24 h after CII stimulation, and found that CII-induced migration and invasion were suppressed, indicating the role of CCR5 in promoting phenotypic changes of FLS in RA. Available evidence has demonstrated that ORM1 can bind to CCR5 on macrophage and skeletal muscle cells [18]. Intriguingly, we observed that knockdown of ORM1 inhibited the expression of CCR5 in CII-induced FLS and CIA rats. These results indicated that ORM1 bound to the CCR5 and positively regulated the CCR5 expression. In parallel with the effect of ORM1 knockdown on CIA rats, CCR5 knockdown was found to inhibit the progression of RA in CIA rats. Collectively, it is believed that ORM1 in combination with CCR5 facilitates the phenotypic change of FLS and the expansion of synovium, leading to cartilage and bone destruction in RA.

Although our study demonstrated a critical role of ORM1-CCR5 axis in promoting RA progression in CII-stimulated FLS and in CIA rat model, we did not perform a direct correlation analysis between ORM1 levels in human serum/synovial fluid and clinical disease activity (e.g., DAS28). This represents an important limitation that will be the focus of our future clinical translational research. It is worth noting that the urinary ORM1 levels in RA patients have a positive correlation with the status of the disease activity [36]. In addition, ORM1 in serum is significantly differentially expressed in normal and RA patient samples, which is positively correlated with disease activity [25]. The existing evidence supported the clinical relevance of ORM1 and the disease activity of RA, necessitating further investigation. Future studies will prioritize the collection of clinical samples from RA patients to definitively establish the relationship between circulating ORM1 levels, synovial tissue expression, and standardized clinical metrics. Furthermore, efforts will be directed toward identifying or developing specific ORM1 inhibitors to fully evaluate the therapeutic potential of targeting this novel pathway in RA.

## Conclusions

In summary, this study provides a new mechanism regulating biological behaviors of FLS in the development of RA. Our current findings suggest that downregulation of ORM1 suppresses the aggressive phenotype of FLS by decreasing the expression of CCR5 to inhibit the progression of RA. Therefore, targeting ORM1 may be a potential strategy for RA management.
